# Generating Pedestrian Trajectories Consistent with the Fundamental Diagram Based on Physiological and Psychological Factors

**DOI:** 10.1371/journal.pone.0117856

**Published:** 2015-04-13

**Authors:** Sahil Narang, Andrew Best, Sean Curtis, Dinesh Manocha

**Affiliations:** 1 Department of Computer Science, University of North Carolina at Chapel Hill, North Carolina, USA; University of Zurich, SWITZERLAND

## Abstract

Pedestrian crowds often have been modeled as many-particle system including microscopic multi-agent simulators. One of the key challenges is to unearth governing principles that can model pedestrian movement, and use them to reproduce paths and behaviors that are frequently observed in human crowds. To that effect, we present a novel crowd simulation algorithm that generates pedestrian trajectories that exhibit the speed-density relationships expressed by the Fundamental Diagram. Our approach is based on biomechanical principles and psychological factors. The overall formulation results in better utilization of free space by the pedestrians and can be easily combined with well-known multi-agent simulation techniques with little computational overhead. We are able to generate human-like dense crowd behaviors in large indoor and outdoor environments and validate the results with captured real-world crowd trajectories.

## Introduction

The problem of simulating the movement and behaviors of human-like crowds is important in many applications, including architecture and urban design, pedestrian dynamics, computer animation, games, virtual reality, etc. In the absence of governing mathematical models, intuition and observations have driven much of the research and development in this field. A key observation in understanding how individual trajectories are formulated arises from studies in pedestrian dynamics and traffic management that highlight the relationship between crowd density and pedestrian movement; as density increases, speed decreases [1–7]. This phenomenon is called the *Fundamental Diagram* [8].

Although real-world pedestrians tend to slow down in areas of high crowd densities, some of the well known crowd simulation models do not exhibit such behaviors. In this paper, we address the problem of modeling crowd behaviors governed by these densities: which we term as *density-dependent behaviors*. In particular, we present a novel approach that combines physiological and psychological principles and effectively generates pedestrian trajectories that exhibit the Fundamental Diagram.

Crowd simulation has been extensively studied and a variety of techniques and models have been proposed to generate plausible, human-like crowd behaviors. Cellular Automate (CA) are some of the oldest approaches for crowd simulation. In CA the workspace of agents is divided into discrete grid cells which can be occupied by at most one agent. Agents then follow simple rules to move towards their goals through adjacent grid cells [9–12]. Continuum methods such as [13–16] model the macroscopic or overall motion of the crowd. Agent-based approaches model each individual in the crowd and the interactions between them [17–22]. The goal of our work is to accurately simulate microscopic agent-agent and agent-obstacle interactions. Therefore, we propose a multi-agent simulation algorithm that specifically models the trajectory and velocity of each individual agent (or particle) over time.

Many prior multi-agent simulation algorithms decompose the trajectory computation problem into two phases: global planning and local navigation. The global planner computes a path through the environment towards the current goal position of each agent while avoiding collisions with the static obstacles. The local navigation techniques take into account the path computed via the global planner and locally modify the trajectory to avoid collisions with dynamic obstacles or other agents in the environment. Collectively, this class of simulators will be referred to as Global-Local Planners (GLP) and such combinations are frequently used to simulate crowd movement and other emergent behaviors [18, 20–28].

Global trajectory planning includes a number of methods including navigation meshes [29], roadmaps and potential fields [13, 30–32], etc. These data structures are computed off-line and then used at run-time to generate collision-free paths around static obstacles in the environment. Some work has been done to adapt these structures to dynamic obstacles [33–35]. Other techniques instead aim to model congestion at the global level and compute paths that simply try to avoid these dense regions [19, 36, 37]. There is extensive literature on local navigation methods that can be used as part of GLP simulators. They include cellular automata [9], force-based [18, 20, 21], rules-based [23], vision-based [24], and velocity-space-based [22, 25, 26] techniques. Recent models handle congestion by using global data structures for path computation [38] or physical forces [39].

Some prior crowd simulation algorithms also seek to model trajectories that are consistent with the Fundamental Diagram. CA models, in particular, are well suited for density computations given the discretization of space. Kretz. at al. [10] describe a probabilistic CA in which agents take into account the density in neighboring cells while choosing their destination cell. Schadschneider et al. [40] focus on calibration of cellular automata models with emperical data by means of the Fundamental Diagram. The authors advocate an extension to the nearest-neighbor restriction in floor field models. There has also been some recent work using Agent-based approaches. Lemercier et al. [41] focus on generating realistic following behaviors based on varying densities. Other algorithms tend to explore the density response at a “meso-scale” level between the global and local planners [42, 43]. In each case, the authors apply local planning techniques to adapt an agent’s path to the one computed to avoid collisions with distant obstacles. The notion of crowd densities and pedestrian motion has also been studied in other disciplines. Biomechanists have studied both personal space and the human gait at an individual level [1, 2]. There is extensive work in pedestrian dynamics on exploring the relationship between speed and density [4–7, 38]. Our approach builds on many of these ideas.

Main Results

We present a novel approach for crowd trajectory computation in dense scenarios that can generate realistic density-dependent behaviors and result in good space utilization. Overall, our approach offers the following benefits:

- The speed and density relationship in the crowd movements computed by our algorithm exhibits the Fundamental Diagram.
- Our approach uses physiological and psychological techniques to model the underlying factors that affect pedestrian movement.
- Our approach results in smoother trajectories and also reduces the number of collisions between the agents.
- Our approach is general and does not make any assumptions about the global planning algorithm or local navigation technique. We have combined our approach with local navigation algorithms based on reciprocal velocity obstacles and social forces.
- The computational overhead of our algorithm is low and we can simulate thousands of agents at interactive rates on a single core.

We validate our approach by comparing with captured crowd trajectories and also demonstrate its performance in indoor and outdoor environments.

A preliminary version of this paper appeared in [44]: this is the full version. The rest of the paper is organized as follows: We describe our approach to crowd simulation and density-dependent behaviors in Section 2. In Section 3, we compare simulation results with the empirical data. We highlight the performance of our algorithm in Section 4. Finally, in Section 5 we analyze the model’s strengths and weaknesses.

## Density-dependent Behaviors

In this section, we describe our approach to introducing density-dependent behaviours into well-known crowd simulation models. First, we introduce some of the notation and terminology used in the rest of the paper. In our crowd simulation algorithm, agents are modeled as two-dimensional disks with the following common *state*: ${\left[r\;\vec{p}\;{\vec{v}}^{c}\;{\vec{v}}^{0}\right]}^{T}\in {\mathbb{R}}^{7}$, where *r* is the radius of the disk, $\vec{p}$, ${\vec{v}}^{c}$, and ${\vec{v}}^{0}$ are two-dimensional vectors representing the current position, current velocity and the *input preferred velocity* generated by the global planner, respectively. By convention, $∥{\vec{v}}^{c}∥$ and $∥{\vec{v}}^{0}∥$ are the current speed and the input preferred speed (i.e. the magnitudes of the corresponding vectors). We use subscripts to denote a particular agent *i*, such as *r*_*i*_ and ${\vec{v}}_{i}^{0}$.

### 2.1 Fundamental Diagram

**Fig 1:**
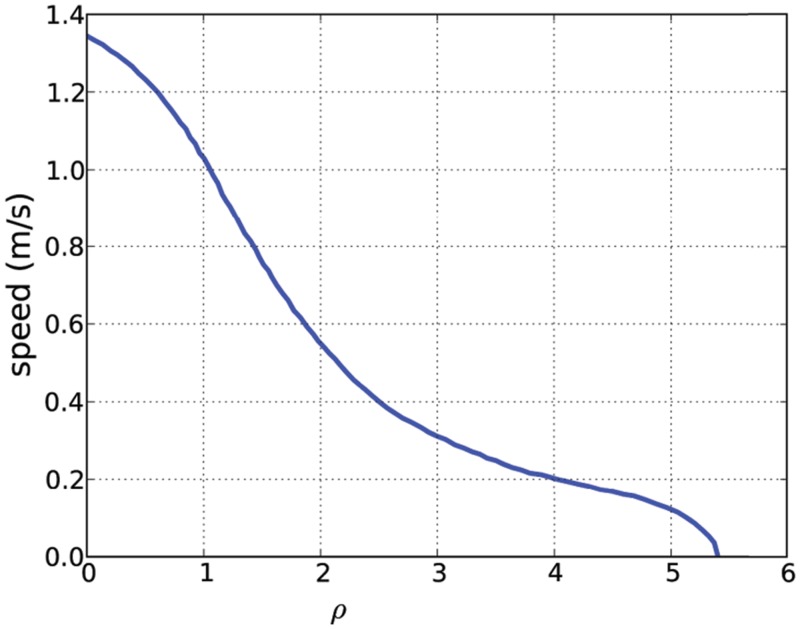
Fundamental Diagram. Empirical relation between pedestrian density and velocity according to Weidmann [8].

The Fundamental Diagram is the observed relationship between pedestrian speed and density; as density increases, speed decreases (Fig 1). Many prior crowd simulation algorithms tend to reduce the speed of the agents through bottlenecks [19, 20]. However, this is largely a function of performing collision avoidance in a congested environment i.e. agents slow down since they cannot move collision-free at a higher speed. It is not a manifestation of the Fundamental Diagram which does not depend on the capacity of a space; it corresponds to a relationship between the density and speed. To that effect, our algorithm aims to slow down agents in accordance with local density even if the agents could possibly move collision-free at a higher speed.

#### 2.1.1 Physiological and Psychological Factors

Our model incorporates a physiological and a psychological factor. The physiological factor is based on the biomechanical principle that stride length and walking speed are inextricably linked; a change in one implies a change in the other [1]. The relationship, as defined by biomechanists [2], is defined by the “stride factor” parameter (*α*). It relates a person’s stride length (*L*) and walking speed (*v*) as:

$$\begin{array}{c} L\left(v\right)=\frac{H}{\alpha }\sqrt{v}, \end{array}$$ (1)

where *H* = *height*/1.72 m is a height-normalizing constant.

The psychological factor models the complex concept of personal space. Psychologists and biomechanists have shown that humans have a strong sense of personal space—a “buffer” that extends beyond mere physical requirements [3, 5]. Our model includes a “stride buffer” parameter (*β*) that reflects this preference for personal space. As the stride buffer increases, an agent requires more space to walk comfortably at a given speed.

We use a linear combination of the physiological and psychological factors to yield a function that determines the natural walking speed (*V*) for the available space (*S*) in front of an agent:

$$\begin{array}{c} V\left(S\right)=\min(∥{\vec{v}}^{0}∥,\;{\left(\frac{S\alpha }{H(1+\beta )}\right)}^{2}), \end{array}$$ (2)

where *α* is the stride factor as defined by [2] and *β* is the stride buffer.

**Space-estimation**: The relationship in Eq 2 is expressed in terms of the space on a line extending in a single direction. However, our agents move on a 2D plane, and we need to compute a similar mapping in a 2D region that comprises of other agents and obstacles. We base our method on some experimental observations performed by Seyfried et al. [4].

Seyfried et al. performed one dimensional experiments on the Fundamental Diagram by creating a narrow passage which constrained the subjects to walk in a line. The subjects’ changes in speed are thus attributed to the available space in front and behind the agent, along the one-dimensional path. The density in this experiment was, likewise, a one-dimensional quantity (number of pedestrians/meter). The authors observed that the 1D density in the experiment was related to the 2D expectations defined by Weidmann [8] by a scale factor equal to the inverse of the individual’s width. Given the average human width of 0.48 m, a measured 1D density value of 1 people/m translates to a 2D density value of 2.08 people/m^2^. We employ this observation in our model of space estimation by first computing the local 2D density and then using Seyfried et. al.’s method to map it to the scalar “space” value used in Eq (2).

### 2.2 Density Dependent Filters

We introduce density-dependent filters that can be used to generate human-like density responses. These filters can be integrated with any Global-Local Planner (GLP) based model.

GLP based models use a global planner to plan a path through the static environment. The path is used to generate immediate goals which are communicated to the local planner as *preferred velocities*—velocities in the direction of an immediate goal, at a user-defined *preferred speed*. The local navigation scheme selects a viable velocity based on the preferred velocity and the local dynamic conditions (i.e. nearby obstacles and agents). In most crowd simulation algorithms based on the GLP paradigm, the preferred velocity serves as the interface between the global and local modules.

Implicit to this model is the assumption that the agent’s global plan is independent of the local conditions. This assumption places a particular burden on the local navigation module; it must be able to compute an appropriate response under an arbitrarily large range of local conditions. This burden can lead to problematic results: noisy trajectories, increased agent-agent collisions and undesirable agent behavior.

**Fig 2:**
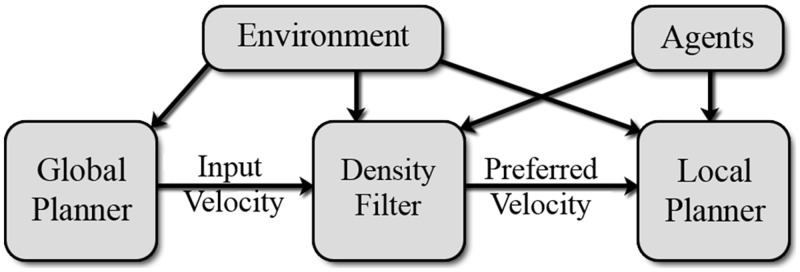
System Architecture. Our density filters act as an interface between the global and local planner.

The density-dependent filters seek to reduce the mismatch between the two stages by seamlessly adapting the preferred velocities generated by the global planner to local dynamic conditions. Architecturally, they serve as the interface between the global planner and local navigation module, as shown in Fig 2. This results in smoother trajectories, reduced agent-agent collisions and realistic agent behavior.

The agents seek to optimize their progress towards the goal by exploring the space around them, while their motion is governed by the physiological and psychological factors. At each step, our algorithm computes an arc centered at the *input preferred velocity* from the global planner. It chooses a new velocity, the *corrected preferred velocity*, from this arc such that it minimizes the cost function (Eq 9) while respecting the physiological and psychological factors that govern pedestrian movement. Finally, the density filter communicates the corrected preferred velocity for collision avoidance.

#### 2.2.1 Agent Density Computation

Here, we describe our approach for computing the contribution of neighboring agents to the density perceived by an agent in the simulation. Given an agent *i* at position ${\vec{p}}_{i}$ and direction vector ${\hat{v}}_{i}^{\theta }$ offset from the agent’s input preferred velocity ${\vec{v}}_{i}^{0}$ by an angle *θ*, we define a point $q_{i}^{\theta }$ that lies one meter ahead along ${\hat{v}}_{i}^{\theta }$. An agent *j* at position ${\vec{p}}_{j}$ is considered to a neighbor of agent *i* if the Euclidean between ${\vec{p}}_{i}$ & ${\vec{p}}_{j}$ is less than a predefined threshold. We use a Gaussian density function to determine the contribution of each neighboring agent to the density at ${\vec{q}}_{i}^{\theta }$. It is well known that humans exhibit an elliptical personal space, with a preference for space in front [3]. To model the personal space of agent *i*, we transform the displacement towards a nearby agent *j* from ${\vec{d}}_{ij}={\vec{p}}_{i}-{\vec{p}}_{j}$ to ${\vec{d}}_{ij_{\theta }}^{\prime }$ causing agents in front to have a greater contribution to density than those to the side (Eqs 5 and 6). Thus, the density of neighboring agents at ${\vec{q}}_{i}^{\theta }$ is:

$$\begin{array}{ccc} \rho_{Ai}^{\theta } & = & \sum_{j}\frac{1}{\sqrt{2\pi \sigma }}e^{\frac{{∥{\vec{d}}_{ij_{\theta }}^{\prime }∥}^{2}}{2\sigma^{2}}}\;j\neq i, \end{array}$$ (3)

$$\begin{array}{ccc} {\vec{q}}_{i}^{\theta } & = & {\vec{p}}_{i}+{\hat{v}}_{i}^{\theta }, \end{array}$$ (4)

$$\begin{array}{ccc} {\vec{d}}_{ij_{\theta }}^{\prime } & = & 2.5\left({\vec{d}}_{ij}-{\vec{d}}_{ij_{\theta }}^{y}\right)+{\vec{d}}_{ij_{\theta }}^{y}, \end{array}$$ (5)

$$\begin{array}{ccc} {\vec{d}}_{ij_{\theta }}^{y} & = & \left({\vec{d}}_{ij}\cdot {\hat{v}}_{i}^{\theta }\right){\hat{v}}_{i}^{\theta }. \end{array}$$ (6)

#### Effect of Obstacles on Density Computation

Here, we describe how obstacles affect the density perceived by an agent. We believe that there exists some discomfort perceived by humans when walking alongside obstacles. We use the inverse of contiguous free space (FS) available to an agent to reflect this discomfort. Essentially, the obstacles are forbidden spaces i.e. an agent may never intersect with an obstacle. At the same time, the obstacles also have a discomfort associated with them that inclines agents to keep a comfortable distance whenever possible.

Given a point $\vec{q}$, we represent this bias using a normalized 2D Gaussian kernel centered at $\vec{q}$. The 2D kernel prioritizes space nearest $\vec{q}$, increasing the agent’s sensitivity to immediately proximate obstacles. The kernel is simply a weight function with compact support (*w*). Integrating it over the domain of the support (Ω) yields the contiguous free space (FS) available to an agent at $\vec{q}$.

$$\begin{array}{c} FS_{q}=\int_{\Omega }w\left(\vec{x}\right) \end{array}$$ (7)

The effective density used in our algorithm for a point ${\vec{q}}_{i}^{\theta }$ is the agent density $\rho_{Ai}^{\theta }$ scaled by the inverse of the free space, $FS(q_{i}^{\theta })$, available at ${\vec{q}}_{i}^{\theta }$.

$$\begin{array}{c} \rho_{i}^{\theta }=\frac{1}{FS({\vec{q}}_{i}^{\theta })}\rho_{Ai}^{\theta } \end{array}$$ (8)

#### 2.2.2 Agent’s Response to Density

**Fig 3:**
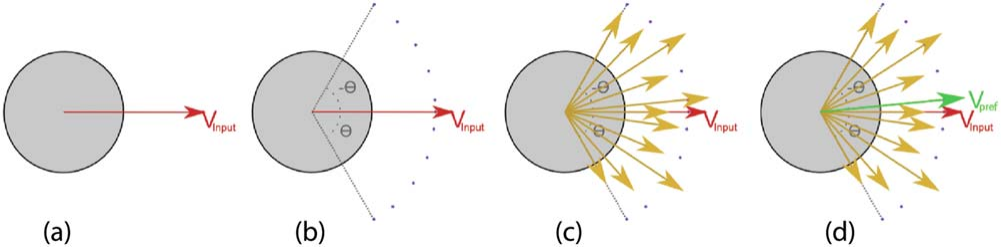
Sampling Mechanism. (a) The input preferred velocity *V*_*input*_ given by the global planner. (b) The agent considers density at critical points along an arc defined by [−*θ*_*max*_, *θ*_*max*_]. (c) It computes the filtered velocities for each critical point along the arc. (c) The velocity which minimizes the cost function, *V*_*pref*_, is chosen as the optimal corrected preferred velocity.

This section describes how an agent computes its *optimal corrected preferred velocity* ${\vec{v}}_{i}^{FD}$. An agent i with width *w*_*i*_ chooses *θ*, the directional offset from its input preferred velocity, such that it minimizes the distance to the goal ${\vec{g}}_{i}$ from its current position ${\vec{p}}_{i}$ with respect to time period *τ* (Fig 3)

$$\begin{array}{c} \arg\min_{\theta }∥\;{\vec{g}}_{i}-\left({\vec{p}}_{i}+{\vec{v}}_{i}^{FD_{\theta }}\tau \right)∥, \end{array}$$ (9)

where ${\vec{v}}_{i}^{FD_{\theta }}$ represents the corrected preferred velocity in direction *θ* that accounts for physiological and psychological constraints. It can be computed as:

$$\begin{array}{c} {\vec{v}}_{i}^{FD_{\theta }}=\frac{{\vec{v}}_{i}^{\theta }}{∥{\vec{v}}_{i}^{\theta }∥}V\left(w_{i}/\rho_{i}^{\theta }\right). \end{array}$$ (10)

The use of the corrected preferred velocity ${\vec{v}}_{i}^{FD_{\theta }}$ in the cost function (Eq 9) implies that an agent implicitly minimizes the time to reach the goal which is consistent with earlier work in pedestrian dynamics [31, 45]. This means that an agent may choose to traverse a longer but less congested path to the goal if it determines it can make more progress towards the goal along that path in the next time step.

### 2.3 Implementation

**Fig 4:**
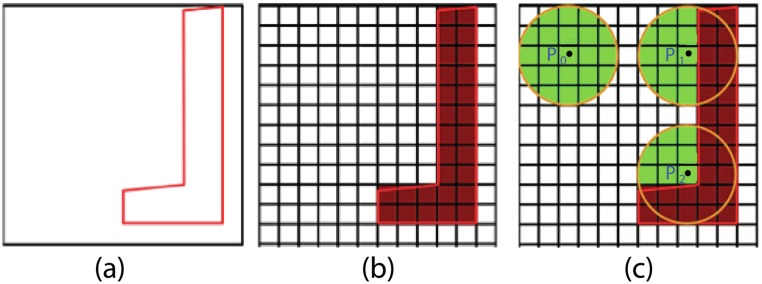
Computing free space at a point. (a) Scene with obstacle (b) Discretized scene (c) Contiguous free space (in green) available to agents centered at points *p*_0_, *p*_1_ and *p*_2_.

We represent the obstacles over a grid of cell size *c*_*b*_. The simulator identifies cells lying inside the obstacle and generates a level set. Next, it computes the free space available at each cell *c*_*p*_ that is not contained within any obstacle. It does so by splatting a precomputed 2D Gaussian kernel at point $\vec{p}$, the center of cell *c*_*p*_ and then systematically walking around the neighborhood of *c*_*p*_ to determine contiguous free space (*FS*) with respect to $\vec{p}$ (Fig 4). Hence, for a scene with *n* obstacles, we can find the obstacles that contribute to “density computation” at a point $\vec{p}$ in *O*(*n*) time. This can all be done offline thereby saving runtime costs.

Algorithm 1 in S1 Table describes the simulation loop for each agent to determine its optimal corrected preferred velocity ${\vec{v}}_{i}^{FD}$. The *DensityAtPoint* and *CorrectedVelocity* procedures referred in Algorithm 1 are described in S2 Table and S3 Table respectively. Finally, the optimal corrected preferred velocity ${\vec{v}}_{i}^{FD}$ is communicated to the local planner for collision avoidance.

### 2.4 Models Augmented with Density Dependent Filters

Our use of density-dependent filters is orthogonal to the choice of global and local planners. To illustrate this generality, as well as our algorithm’s efficacy, we have applied it to several local planners. The simulators all share the same global planner, which is a navigation mesh. The shared global planner means the differences between simulators will be dominated by the local planners. In particular, we demonstrate how our approach can be combined with two widely use local navigation algorithms: social forces [20] and optimal reciprocal collision avoidance [22]. It is important to note that the time period *τ* used in Eq 9 should be greater than the time step for either of the local planners. This implies that we optimize the preferred velocity for a larger time scale but account for collision avoidance on a smaller time scale.

#### 2.4.1 Social Forces

The social force (SF) model treats the crowd as a collection of mass particles. Newtonian-like physics is applied to the system to compute the trajectories of the agents. At each time step, the superposition of various forces are computed for each agent, ultimately determining a feasible velocity by imparting acceleration on the agent. The preferred velocity is converted into a “driving force”. The agent avoids collisions with other agents and obstacles through the application of repulsive forces. We have taken the exact formulation of these forces, including parameter values, from Helbing’s work [20].

Social Forces with Density Dependent Filters (D-SF): An agent *i* under D-SF feels a driving force ${\vec{F}}_{drive}$ as:

$$\begin{array}{c} {\vec{F}}_{drive}=m_{i}\frac{{\vec{v}}_{i}^{FD}-{\vec{v}}_{i}^{new}}{\tau^{H}}, \end{array}$$ (11)

where *m*_*i*_ is the mass of the agent, *τ* ^*H*^ is the time step for the numerical solver and ${\vec{v}}_{i}^{new}$ is the new velocity for agent *i*. ${\vec{v}}_{i}^{new}$ can be computed using the force equation:

$$\begin{array}{c} m_{i}\frac{d{\vec{v}}_{i}^{new}}{dt}={\vec{F}}_{drive}+\sum_{j\neq i}{\vec{f}}_{ij}+\sum_{W}{\vec{f}}_{iW}, \end{array}$$ (12)

where ${\vec{f}}_{ij}$ represents the agent-agent repulsive force and ${\vec{f}}_{iW}$ the agent-obstacle repulsive force.

#### 2.4.2 Optimal Reciprocal Collision Avoidance

Optimal Reciprocal Collision Avoidance (ORCA) [22] applies geometric optimization techniques in velocity space. It is based on the notion of velocity obstacles from robotics. Avoiding collision simply requires selecting a velocity which does not lie within the reciprocal velocity obstacle set for each agent. ORCA’s unique formulation defines the velocity obstacles as half planes. The set of velocities that are permitted for agent *i* with respect to all other agents is the intersection of the half planes of permitted velocities induced by each other agent, and we denote this set ${ORCA}_{i}^{\tau^{O}}$ for the planning horizon *τ* ^*O*^.

ORCA with Density Dependent Filters (D-ORCA): In the D-ORCA algorithm, an agent selects a new velocity ${\vec{v}}_{i}^{new}$ for itself that is closest to its optimal corrected preferred velocity ${\vec{v}}_{i}^{FD}$ amongst all velocities inside the region of permitted velocities:

$$\begin{array}{c} {\vec{v}}_{i}^{new}=\arg\min_{\vec{v}\in {ORCA}_{i}^{\tau^{O}}}∥\;\vec{v}-{\vec{v}}_{i}^{FD}∥ \end{array}$$ (13)

## Comparison to Real-World Data

**Fig 5:**
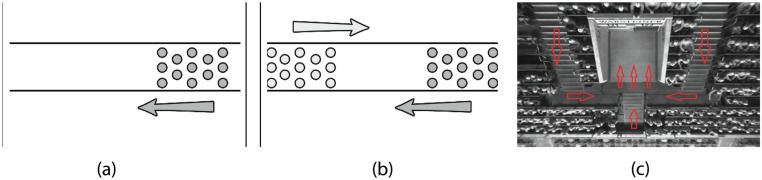
Benchmark scenarios used to validate our density dependent filters. (a) The uni-directional flow experiment in [6] (c) The bi-directional flow experiment in [7] and (c) The stadium experiment [46] with red arrowheads denoting the path to the exit tunnel.

We validate the performance of our model in three reproductions of live pedestrian experiments (Fig 5). These real world experiments examine the flow of pedestrians in various scenarios: a 2D uni-directional flow scenario, a bidirectional flow scenario, and a complex flow scenario of people exiting a stadium. For each experiment, we report adherence to the Fundamental Diagram.

**Fig 6:**
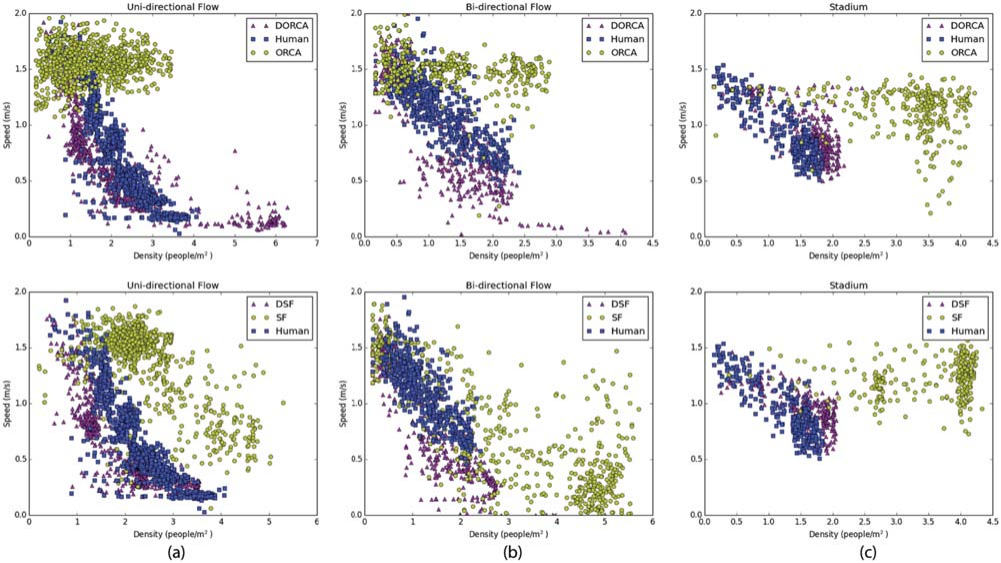
Fundamental Diagram for SF, D-SF, ORCA, D-ORCA for the three experiments. (a) The uni-directional 2D flow experiment in [6] (b) The bi-directional flow experiment in [7] and (c) The stadium experiment [46]. Agents in both D-ORCA and D-SF exhibit a human-like sensitivity to high densities and conform to the Fundamental Diagram.

We measure the simulator’s ability to reproduce the Fundamental Diagram in the following manner. For each experiment, we define a region through which each agent must pass. We compute the time it takes for each agent to pass through the region and the average density of the agents in that region during that interval. This becomes a single density-speed data pair, which we present in a scatter plot (Fig 6). This analysis is consistent with earlier results reported in the pedestrian dynamics literature [4–7].

When the flow of agents is reduced because the capacity of the area is limited, congestion increases and the agents slow down. It is worth noting that both SF and ORCA agents exhibit such behavior. However, this is not a manifestation of the Fundamental Diagram. The Fundamental Diagram does not require changes in the capacity of a space. Simply increasing density should cause a reduction in speed.

### 3.1 Two Dimensional, Uni-Directional Flow

Zhang et al. [6] performed experiments of uni-directional flow in a corridor (Fig 5(a)). The authors performed multiple iterations of the experiment, varying the flow into the corridor and the flow out of the corridor to control the observed density in the measurement region inside the corridor. Reproducing this experiment tests the suitability of the space-estimation formulation described earlier.

Fig 6(a) shows the Fundamental Diagram results for ORCA, D-ORCA, SF and D-SF. Its easy to see that both ORCA and SF marginally reduce speeds at high densities. This is due to the constraints on the flow out of the corridor. The experiment created a bottleneck; the flow into the corridor was greater than the flow out of the corridor. However, D-ORCA and D-SF agents reduce speeds due to increase in density which is in accordance with the Fundamental Diagram and closely matches the captured human data.

### 3.2 Two-dimensional, Bi-directional Flow

Following the uni-directional flow experiment, Zhang et al. also examined bi-directional flow [7]. Using the same arrangement as in the previous uni-directional flow experiment, the authors placed subjects at both ends of the corridor. In our bi-directional experiment, we use an infinitely long corridor, of the same width in the pedestrian experiment. We vary the flow into the corridor by varying the initial agent density (ranging from 0.5–2.5 people/m^2^). The Fundamental Diagram results can be seen in Fig 6(b). SF showed a consistent reduction in speed with respect to density. In contrast, ORCA exhibited relatively constant speeds irrespective of density. However, with the application of the density filters, agents were able to maintain a sparse distribution, which appears to be consistent with observed human-behaviors.

### 3.3 Stadium

Burghardt et. al. [46] performed experiments on the complex flow of a crowd exiting a soccer stadium. They recorded the trajectories of 300 spectators as they made their way toward a predetermined exit tunnel. The stadium is difficult to simulate because of its non planar layout; furthermore, there are three dense flows that meet almost orthogonally at the entrance of the tunnel which leads to a bottleneck. Fig 6(c) depicts the Fundamental Diagram, computed at the entrance of the tunnel, for this dataset. It is easy to see the success of the density filters at effectively modeling the highly dense and convoluted flows that make up the stadium dataset. In contrast, both ORCA and SF agents achieve significantly high densities and exhibit little or no response to density.

**Fig 7:**
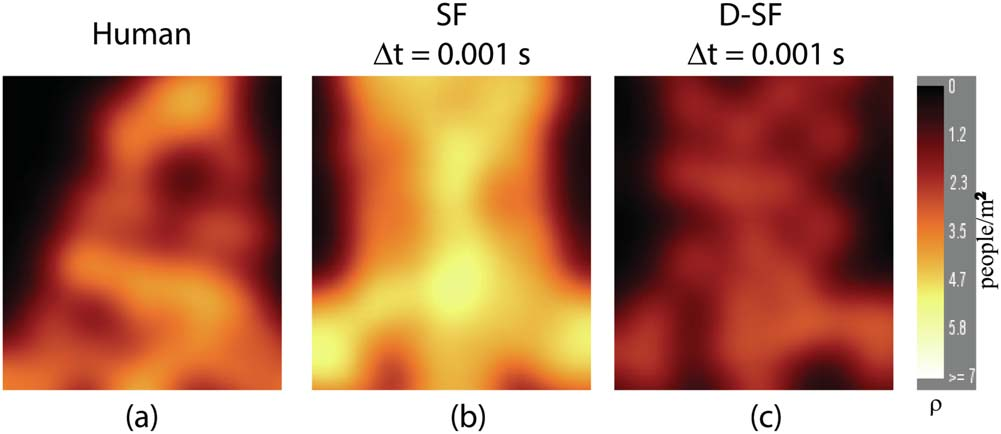
Density visualization for stadium experiment [46]. a) Captured pedestrian crowd b) SF and c) D-SF at the moment when 20% of the agents have entered the exit tunnel. D-SF agents maintain a sparse distribution, similar to captured pedestrian crowd while SF agents achieve densities as high as 5.93 people/m^2^.

D-SF’s response to density is further evident from the density field for captured pedestrian crowd (Fig 7(a)), D-SF (Fig 7(b)), and SF (Fig 7(c)) in the exit tunnel at the moment when 20% of the agents have entered the tunnel. SF agents achieve densities as high as 5.93 people/m^2^ whereas D-SF agents achieve a maximum value of 3.96 people/m^2^, more in keeping with captured human data.

## Results

In this section, we highlight the performance of our algorithm on different scenarios. In addition to generating density-dependent behaviors, our formulation also results in smoother trajectories and results in fewer agent-agent collisions as compared to prior methods.

### 4.1 Applications

**Fig 8:**
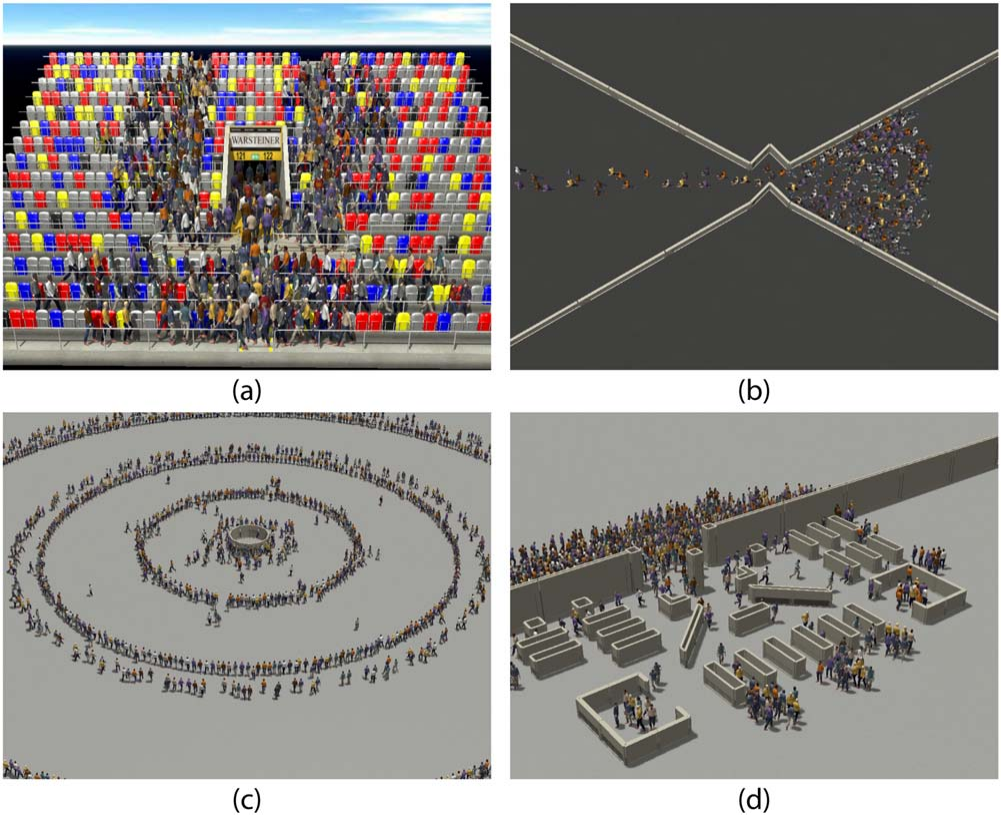
Application scenarios with D-ORCA agents. a) Stadium b) Hourglass c) Ticket Kiosk and d) Shopping Mall.

We tested the capabilities of our model on a number of example scenarios, depicted in Fig 8. In one scenario, roughly depicting an hourglass, 200 agents stream in towards a narrow passage with concave windings (Fig 8(b)). Agents under D-SF and D-ORCA exhibit significantly fewer collisions than their SF and ORCA counterparts. In another scenario, agents move in concentric circles towards a ticket kiosk at the center (Fig 8(c)). They are programmed to wait for 10 seconds at the kiosk before heading outward again. This creates a high density situation since we have a large number of agents heading towards each other with opposing goals. Agents under D-SF and D-ORCA exhibit smoother trajectories and are able to reach their goals faster as compared to SF and ORCA respectively. Finally, we depict a dense crowd entering a shopping mall(Fig 8(d)). One thousand agents queue up outside the store waiting for the doors to open. Once opened, agents stream in and move towards the other end of the store. D-SF and D-ORCA agents exhibit a tendency to maintain personal space and to this end, utilize the free space around them. This translates to smoother trajectories and few collisions as compared to SF and ORCA respectively.

It is worth noting that we used the same sampling parameters for all our experiments. Furthermore, with the obstacle grid computed offline, the run time overhead of our algorithm was minimal, as can be seen in Table 1. In most cases, the introduction of the density filters increased the average frame computation time by a few milliseconds. However, in case of the Kiosk, D-SF actually reduced the computation time. This is due to the significant decrease in collisions (Table 2). The experiment created cross flows leading to regions of high density. SF agents attempted to achieve their preferred speed even in high densities, leading to collisions and thus increasing the time spent in collision detection. On the other hand, D-SF agents were able to maintain a sparse distribution and thus had fewer collisions.

**Table 1:** Average frame computation time.

**Scenario**	**Agents**	**ORCA (ms)**		**SF (ms)**	
		ORCA	D-ORCA	SF	D-SF
Stadium	302	2.10	13.27	2.32	15.02
Hourglass	200	1.15	12.31	1.01	12.07
Kiosk	1830	17.05	71.51	33.81	10.17
Shopping Mall	980	11.55	111.49	11.29	139.70

Application of the density filters only marginally increases the frame computation time. In case of the Kiosk, it actually reduced the computation time due to a significant decrease in collisions.

**Table 2:** The impact of the density filters on collisions.

**Scenario**	**ORCA**		**SF**	
	ORCA	D-ORCA	SF	D-SF
Uni-dir.	0.07	1.5E-03	0.181	0
Bi-dir.	0.526	0	0.52	3.07E-04
Stadium	0.112	9.6E-03	0.233	7.3E-05
Hourglass	0.841	4.19E-04	0.921	0
Kiosk	1.447	0.508	4.681	4.1E-03
Shopping Mall	1.327	0.112	11.051	6.4E-03

Collision scores were computed using the interval penetration depth metric, described in 4.2. Application of the density filters considerably reduces the collision rate for both simulators.

### 4.2 Inter-agent Collisions

Finally, models augmented with density-dependent filters reduce the number and degree of collisions in the simulation. By a collision, we mean the overlap or penetration of one agent into the geometric shape corresponding to another agent. We use *interval penetration depth* as a metric for measuring the rate of collisions in a crowd.

Penetration depth is the measure of how much two entities overlap; it is typically defined as the minimum displacement required to eliminate overlap. It allows us to quantify the *severity* of collision. For each pair of adjacent time steps, we compute the penetration depth between two agents, *i* and *j*, over the interval bound by those time steps *t* and *t* + 1. Finally, we produce a collision score for a full simulation by computing the average penetration depth across all frames and agents.

Table 2 reports the collision scores for the scenarios, with and without the density filters. The order of magnitude difference in logarithmic scale with the application of the density filters is high as ten for both ORCA and SF. The “Bidirectional flow” in case of ORCA and “Hourglass” scenario in case of SF witnessed the most improvement which is understandable since these scenarios were designed to have the most cross flow. The fact that our density filter reduces collisions should not be surprising. Many of the collisions arise from the jostling due to non-smooth trajectories in dense scenarios. Smoother trajectories naturally lead to fewer collisions.

### 4.3 Trajectory Smoothness

Without the density filter, the trajectories exhibit a great deal of noise. The reason for this is that the unfiltered intention (preferred speed) might be inconsistent with the agents current condition; moving toward the goal at preferred speed may not be possible. But given that intention, the local navigation algorithm aggressively seeks to exploit every opportunity to increase the agent’s speed to match its preferred speed. However, such rapid accelerations can often leave the agent in an untenable situation which needs to be reversed in the very next time step. The introduction of the density filter causes the intended speed to scale down according to the congestion. Because of this, the acceleration taken is more likely to be valid over a longer window and the motion of the agent becomes smoother.

**Fig 9:**
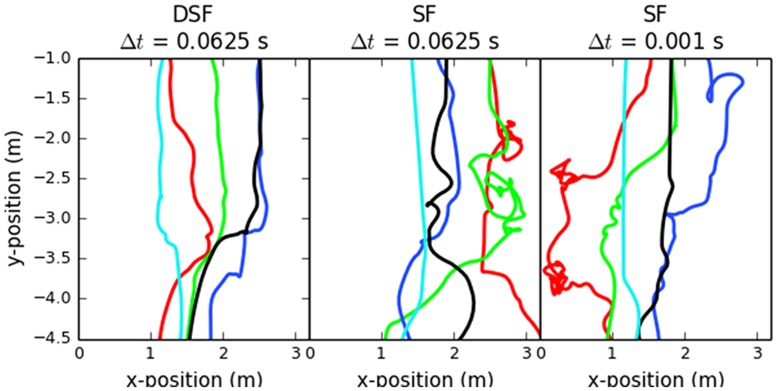
Impact of density filters on trajectory smoothness. Trajectories of five sampled SF agents under three simulation paradigms: with D-SF at 16 Hz (left), SF at 16 Hz (middle), and SF at 1000 Hz (right). D-SF agents exhibited the smoothest trajectories.

We illustrate our model’s impact on trajectory smoothness by plotting trajectories of a small sample of agents (Fig 9). We performed a baseline simulation using SF with a time-step of 0.0625 s (middle). In addition, we simulated D-SF at the same time step (left) and SF at a 0.001 s time step (right). The baseline trajectories are chaotic. Reducing the time step by a factor of 62.5 improved smoothness, but introducing the density filter produced the smoothest trajectories, even at the larger time step.

## Conclusion

We have introduced a novel crowd simulation algorithm based on density-dependent filters to generate human-like crowd flows. Our approach is applicable to a large number of GLP multi-agent algorithms that use a combination of local and global planners, and can generate realistic density-dependent behaviors. We have highlighted the performance on many complex scenarios and also validated the performance with captured human trajectories. The computational overhead of our approach is relatively small, and it is shown to generate smoother trajectories with fewer collisions. Furthermore, the introduction of the density filters requires no significant changes to either the global or local navigation components.

### 5.1 Limitations and Future Work

The potential impact of the density filters depends on how sensitive the local planner is to the preferred velocity. In both SF and ORCA, the preferred velocity plays a significant role and this results in improved performance of D-SF and D-ORCA. Also, the benefit of the density filters may be relatively less in scenarios where global density-dependent navigation techniques are used. The fact that the adaptation is done at a local level implies that the density filters may prove ineffective in scenarios where global density-dependent navigation techniques are more appropriate.

In future work, we will investigate the application of the density filters at the meso-scale so as to allow agents to account for sudden changes in density. Furthermore, we plan to augment our density filters with other psychological factors which may play a vital role in modeling dense heterogeneous crowds.

## Supporting Information

S1 Table **Algorithm 1.** Simulation loop for each agent *i*. (TEX)

S2 Table **Algorithm 2.** Computing density at a point for agent *i*. (TEX)

S3 Table **Algorithm 3.** Corrected preferred velocity for agent *i*. (TEX)
